# Brill-Zinsser Disease in Moroccan Man, France, 2011

**DOI:** 10.3201/eid1801.111057

**Published:** 2012-01

**Authors:** Jean-François Faucher, Cristina Socolovschi, Camille Aubry, Catherine Chirouze, Laurent Hustache-Mathieu, Didier Raoult, Bruno Hoen

**Affiliations:** Besançon University Hospital, Besançon, France (J.-F. Faucher, C. Chirouze, L. Hustache-Mathieu, B. Hoen);; Université de la Méditerranée, Marseille, France (C. Socolovschi, C. Aubry, D. Raoult);; World Health Organization Collaborative Center for Rickettsial Diseases and Other Arthropod-borne Bacterial Diseases, Marseille (C. Socolovschi, C. Aubry, D. Raoult)

**Keywords:** Brill-Zinsser disease, Rickettsia prowazekii, Morocco, France, typhus, bacteria

**To the Editor:** Epidemic typhus is caused by *Rickettsia prowazekii* and transmitted by human body lice. For centuries, it has been associated with overcrowding, cold weather, and poor hygiene. Brill-Zinsser disease is a recurrent form of epidemic typhus that is unrelated to louse infestation and develops sporadically years after the primary illness. Clinical features are similar to, but milder than, those of epidemic typhus (*1*). We report a case of Brill-Zinsser disease in a patient who was born in Morocco and had no history of epidemic typhus.

A 69-year-old man living in France sought care from his general practitioner on March 7, 2011, after 2 days of high-grade fever (40°C) associated with headache, myalgia, fatigue, and mild cough. Amoxicillin was prescribed for a putative diagnosis of acute respiratory infection.

He was admitted to hospital on March 9 for persistent fever. Physical examination results were unremarkable. Blood test results were as follows: C-reactive protein 111 mg/L (reference 0–8 mg/L); procalcitonin 0.49 ng/mL (reference 0.1–0.4 ng/mL), lymphocyte count 0.7 × 10^3^ cells/μL (reference 1–4 × 10^3^ cells/μL), platelet count 92 × 10^3^ cells/μL (reference 150–450 × 10^3^ cells/μL), and lactate dehydrogenase 376 U/L (reference 94–246 U/L). Chest radiograph results were normal. Results of 5 blood cultures and a urine culture were negative. Stupor developed on March 11. Cerebrospinal fluid test results were normal. Because the patient lived near a goat farm, Q fever and tularemia were considered plausible hypotheses, and oral doxycycline was introduced on March 13. The patient became afebrile on March 15, and he was discharged from the hospital and remained well.

On the basis of serologic results, the following diagnoses could be ruled out: viral infections (HIV, cytomegalovirus, Epstein-Barr virus); tularemia; Q fever; leptospirosis; salmonellosis; and *Legionella*, *Mycoplasma*, and *Chlamydia* spp. infections. Acute-phase and convalescent-phase serum samples were positive for typhus-group rickettsiae by the microimmunofluorescence assay at the World Health Organization Collaborative Center for Rickettsioses and Other Arthropod-Borne Bacterial Diseases (Marseille, France). A microimmunofluorescence assay showed titers of 100 for IgM and 6,400 for IgG. Western blot analyses and cross-adsorption studies strongly suggested *R. prowazekii* as the cause of the man’s illness. Quantitative PCR result on DNA extracted from the acute-phase serum was negative (*2*).

The patient had been raised in Morocco. At 19 years of age, he emigrated to France, where he lived in a urban area. He subsequently traveled every 3 years to Morocco for 1-month summer holidays, always in urban areas. He had most recently traveled to Morocco in 2008. He denied any history of hospitalization for a severe febrile illness and any exposure to louse bites. In the weeks before disease onset, he had not taken any new drug. He had no immunoglobulin deficiency.

On the basis of serologic analysis with Western blot, we confirmed *R. prowazekii* infection in a patient with no recent travel and no contact with lice or flying squirrels. *R. prowazekii* infection may occur rarely in France; it was found in Marseille in 2002 in an asymptomatic homeless person (*3*). In contrast, the patient in our report was living in a hygienic environment, and an autochthonous infection is therefore highly unlikely.

Epidemic typhus was endemic to North Africa until the 1970s (*4*). Subsequently, this region was thought to be free from epidemic typhus, but 2 cases have been reported since 1999 in Algeria, where 1 case of Brill-Zinsser disease was observed in a man who had had epidemic typhus in 1960 during the Algerian civil war (*5**–**7*). Few published data exist about the seroprevalence of *R. prowazekii* infections in North Africa (*4*). In Tunisia, no epidemic typhus was found in 2005 among 47 febrile patients (*8*). However, a seroepidemiologic survey performed in blood donors and hospitalized patients in the Aures, Algeria, found a prevalence of 2% (*4*). This finding suggests that *R. prowazekii* infection might have occurred in this population more often than suspected. No recent published data are available from Morocco.

Since 1970, reports of only 8 cases of Brill-Zinsser disease have been published (*9**,**10*). In all cases, known risk factors were present (overcrowding, poor hygiene, or contact with flying squirrels). Brill and Zinsser described that stress or waning immunity could reactivate *R. prowazekii* infection (*2*). Corticosteroids can trigger recurrence of *R. prowazekii* in mice (*2*), but no such observations were made in humans. In the case presented here, we found no stress factor, no immunosuppression, and no medical history of epidemic typhus.

Brill-Zinsser disease can develop >40 years after acute infection. The mechanism of *R. prowazekii* latency has not been established. A recently explored reservoir for silent forms of *R. prowazekii* infection is adipose tissue because it contains endothelial cells, which are the target cells for *R. prowazekii* infection, and because of its wide distribution throughout the body (*2*). Brill-Zinsser disease should be considered as a possible diagnosis for acute fever in any patient who has lived in an area where epidemic typhus is endemic.
